# War Work at the London Hospital

**Published:** 1915-06-05

**Authors:** 


### WAR WORK AT THE LONDON HOSPITAL.

### Staff, Salaries, and Air-raid Decisions.

At the quarterly court of the London Hospital held last Wednesday, the number of wounded soldiers treated since the outbreak of the war was given as 2,688, of which 472 were Belgians. The following interesting facts are contained in the quarterly report:

The arrangements for the drafting and reception of the wounded are now working very well. A daily return of the number of beds available is sent to the War Office, and the War Office give reasonable notice of the sending of any wounded, and also information as to the definite number. It is also satisfactory that the cases which are now being sent to the hospital are of a more serious nature, since it is only reasonable that a hospital fully equipped and staffed with experts as the London should not be used for quite minor cases. Hitherto only thirty deaths have occurred among the soldiers.

### The Staff and the Orderlies.

As the war continues the difficulties as regards the work of the hospital do not diminish. The staff of resident doctors is reduced to a minimum, and the committee wish especially to thank the members of the visiting staff for their generous assistance in making up for the deficiencies of the resident staff. As regards the maternity department, student maternity assistants are unobtainable, and the maternity work of the district, which has hitherto been performed by them, is now carried on by sisters and nurses.

The number of student dressers available is also very greatly reduced, and quite inadequate for the work of the hospital. The committee made arrangements with the R.A.M.C. Training Centre at Crookham to receive and train R.A.M.C. orderlies. Twenty orderlies, under the charge of a lieutenant, are received for one month's training in practical work. The orderlies are allotted to the various wards, the out-patient department, and the receiving room. They are also called up when wounded are sent to the hospital to transfer them from the ambulances to the wards. The scheme has proved a great success. All the orderlies who have been sent have proved very keen to learn the practical side of the work, and the training which the hospital has been able to give them is doubtless of considerable value. On the other hand, they are very useful to the hospital in doing work which has hitherto been performed by dressers. They are quartered at night in the out-patient department, and the War Office pays for their board at the rate of 1s. 9d. per diem per man.

### Expenditure and Salaries.

The committee anticipate that the expenditure this year owing to the general rise in prices of all commodities and wages will show a considerable increase. Drugs and chemicals are particularly affected. The increase in price, for instance, of such a stock commodity as Epsom salts is 50 per cent. Aspirin has increased from 2s. to 27s. per lb. Carbolic acid, moreover, owing to its use in the making of explosives, is now procurable only on the special authority of the War Office. Every effort possible is made to economise in the use of drugs.

As regards wages, the committee, in view of the increased cost of living, decided to grant a war bonus to employees earning less than £150 a year. The bonus has been allocated according to scale, and does not exceed a maximum of 3s. per week. The committee also decided to grant a badge to the regular servants of the hospital, indicating that they are employed at the London Hospital. It was considered that this was advisable since certain of the younger employees have been made the objects of derision owing to not having enlisted.

The committee wish to record special thanks to the Sydney Consignments Committee of the London Chamber of Commerce for valuable gifts of mutton, flour, beef, rabbits, sugar, butter, etc., and also the Queensland Government for similar gifts. These gifts have proved most valuable in lessening the difficulties and the cost of catering for the wounded. The British Red Cross Society has kindly presented to the hospital six well-made and well-sprung stretched trolleys for conveying the wounded from the ambulances to the wards.

The agreement with the London County Council whereby the hospital undertakes to treat 3,000 eye cases, 3,000 throat and ear cases, 350 *x*-ray cases, and 1,540 dental cases, has been renewed for a period of one year from March 31 last. The carrying out of this agreement is not without difficulty, but the committee feel that every effort should be made to assist the Council in the care of the children.

The work of building the salvarsan wards, the cost of which is most generously being defrayed by the Grocers' Company, is proceeding. Originally a German product, salvarsan is now being made by British and French firms.

### Air Raids and Protection Schemes.

With regard to the raid by Zeppelins or aircraft, the German and British Governments have agreed upon a protective sign for churches, museums, hospitals, etc. This sign, which consists of a square divided diagonally with black and white, has been painted on the roof of the hospital. Special instructions have been issued to the sisters and nurses in the event of bombs being dropped in the vicinity of the hospital. As it is possible that bombs may emit poisonous gases, stress has been laid on the necessity of shutting all basement and ground-floor windows, since the gas used would undoubtedly be heavy, and would not rise far from the ground. Certain experiments have also been made with regard to the extinguishing of fires caused by incendiary bombs. These bombs undoubtedly consist largely of phosphorus dissolved in carbon bisulphide, for the extinguishing of which water is more satisfactory than sand.

